# Diagnostic accuracy of radiomics and artificial intelligence models in diagnosing lymph node metastasis in head and neck cancers: a systematic review and meta-analysis

**DOI:** 10.1007/s00234-024-03485-x

**Published:** 2024-11-11

**Authors:** Parya Valizadeh, Payam Jannatdoust, Mohammad-Taha Pahlevan-Fallahy, Amir Hassankhani, Melika Amoukhteh, Sara Bagherieh, Delaram J. Ghadimi, Ali Gholamrezanezhad

**Affiliations:** 1 School of Medicine, Tehran University of Medical Sciences, Tehran, Iran; 2 Department of Radiology, Keck School of Medicine, University of Southern California (USC), 1441 Eastlake Ave Ste 2315, Los Angeles, CA 90089 USA; 3 Department of Radiology, Mayo Clinic, Rochester, MN USA; 4 School of Medicine, Isfahan University of Medical Sciences, Isfahan, Iran; 5 School of Medicine, Shahid Beheshti University of Medical Sciences, Tehran, Iran; 6 Department of Radiology, Los Angeles General Hospital, Los Angeles, CA, USA

**Keywords:** Head and neck cancer, Lymph node metastasis, Radiomics, Deep learning, PET/CT imaging

## Abstract

**Introduction:**

Head and neck cancers are the seventh most common globally, with lymph node metastasis (LNM) being a critical prognostic factor, significantly reducing survival rates. Traditional imaging methods have limitations in accurately diagnosing LNM. This meta-analysis aims to estimate the diagnostic accuracy of Artificial Intelligence (AI) models in detecting LNM in head and neck cancers.

**Methods:**

A systematic search was performed on four databases, looking for studies reporting the diagnostic accuracy of AI models in detecting LNM in head and neck cancers. Methodological quality was assessed using the METRICS tool and meta-analysis was performed using bivariate model in R environment.

**Results:**

23 articles met the inclusion criteria. Due to the absence of external validation in most studies, all analyses were confined to internal validation sets. The meta-analysis revealed a pooled AUC of 91% for CT-based radiomics, 84% for MRI-based radiomics, and 92% for PET/CT-based radiomics. Sensitivity and specificity were highest for PET/CT-based models. The pooled AUC was 92% for deep learning models and 91% for hand-crafted radiomics models. Models based on lymph node features had a pooled AUC of 92%, while those based on primary tumor features had an AUC of 89%. No significant differences were found between deep learning and hand-crafted radiomics models or between lymph node and primary tumor feature-based models.

**Conclusion:**

Radiomics and deep learning models exhibit promising accuracy in diagnosing LNM in head and neck cancers, particularly with PET/CT. Future research should prioritize multicenter studies with external validation to confirm these results and enhance clinical applicability.

**Supplementary Information:**

The online version contains supplementary material available at 10.1007/s00234-024-03485-x.

## Introduction

Head and neck cancers are the seventh most common cancer worldwide and primarily consist of squamous cell carcinomas of the oral cavity and pharynx [1]. According to the Global Burden of Disease Study, their annual mortality rate was estimated to be 313,000 deaths in 2019 [2]. Lymph node metastasis (LNM) is the most critical prognostic factor in head and neck cancers, reducing the survival rate to half [3]. While the treatment strategy depends on the LNM status, there is no consensus on neck dissection or close follow-up in early-stage head and neck cancers [4]. Failing to treat a metastatic lymph node may lead to disease recurrence. Treating a benign lymph node with surgery or radiation, particularly when in close proximity to vital structures, can result in unnecessary side effects and complications for the patient [5].

LNM is typically diagnosed based on its morphological features on imaging. The most commonly used characteristics are size, irregularity, necrosis, cystic degeneration, spherical shape, and clustering lymph nodes [6, 7]. However, enlargement may be observed in reactive lymph nodes, whereas malignant lymph nodes may maintain normal morphology [8]. A meta-analysis found a sensitivity and specificity of 52% and 93% for computed tomography (CT) scan, 65% and 81% for magnetic resonance imaging (MRI), 66% and 87% for positron emission tomography (PET), and 66% and 78% for ultrasonography (US), respectively in diagnosing LNM in head and neck cancers [9]. Meanwhile, in 30% of patients without clinical or radiological evidence of LNM, histopathological examinations show positive lymph node infiltration [10]. As a result, many of the clinically lymph node-negative patients undergo lymph node dissection. Also, not all clinically LNM-positive patients who undergo surgery are histopathologically proven to have LNM, as radiological LNM diagnosis is not thoroughly accurate [11]. There is a considerable chance that a large proportion of patients will receive inaccurate clinical nodal staging.

Besides the inherent difficulties of detecting LNM, like tissue characteristics and technical barriers, the most critical factor in accurate diagnosis is human errors affected by physician experience and busy radiologists’ workflow [12, 13]. Computer-assisted diagnostic systems that integrate qualitative and quantitative imaging features to diagnose LNM might be a solution to enhance diagnostic accuracy and implement personalized treatment. The region of interest (ROI) used for extracting the relevant features might be the lymph nodes or the primary tumoral tissue [14].

Hand-crafted radiomics (HCR) methods extract and analyze a multitude of quantitative features and, with the help of machine learning algorithms, classify a tissue into metastatic or non-metastatic [15, 16]. Also, deep learning algorithms extract relevant features from a picture, transmit them through multiple layers of neural networks, and finally perform classification [17]. In this systematic review and meta-analysis, we intended to estimate the diagnostic accuracy of HCR and deep learning algorithms in detecting LNM of head and neck cancers on different imaging modalities.

## Methods

In adherence to the Preferred Reporting Items for Systematic Reviews and Meta-Analyses (PRISMA) statement guidelines [18], we performed a comprehensive search across PubMed, Scopus, Web of Science, and Embase. We designed a search string for each database, including keywords for (“artificial intelligence” OR “artificial neural networks” OR “machine learning” OR “deep learning” OR “convolutional neural network” OR “automatic detection” OR “radiomic” OR “radiomics”) AND (“computed tomography” OR “computed tomography scan” OR “CT scan”) AND (“lymph node” OR “lymph nodes” OR “nodal” OR “node” OR “metastasis” OR “metastases” OR “lymph node metastasis” OR “lymph node metastases”) AND (“head and neck neoplasm” OR “head and neck cancer” OR “head and neck squamous cell carcinoma” OR “HNSCC”). Moreover, we conducted a manual examination of the references section of the included studies, searching for further relevant papers.

Two researchers independently reviewed each article’s title, abstract, and/or full text and assessed their relevance to the inclusion criteria. In case of any disagreements, a consensus on whether to include the study was reached through consultation with a senior co-author. The AutoLit platform, created by Nested Knowledge in St. Paul, Minnesota, USA, was used to assist with deduplication, screening, and data extraction.

Relevant studies reporting at least one discrimination statistic for radiomics and/or deep learning models were eligible for inclusion. We imposed no limitations regarding the country, study design, year of publication, or patient characteristics. We excluded non-English publications, case reports, and case series with less than five patients, as well as conference abstracts, review articles, and editorial comments. We extracted data such as the first author’s name, year of publication, imaging modality, assessed condition, sample size and demographics, segmentation method, reference test, developed diagnostic models, and the discrimination statistics of the primary model.

In this systematic review, we utilized the METhodological RadiomICs Score (METRICS) checklist to evaluate the quality of the included studies [19]. The METRICS checklist offers a detailed framework for scrutinizing key methodological aspects pertinent to both handcrafted radiomics and deep learning models. The evaluation encompasses several domains, including study design, imaging data, segmentation, image processing and feature extraction, feature processing, preparation for modeling, metrics and comparison, testing, and open science. Each domain comprises multiple questions, each assigned a specific weight, enabling a comprehensive assessment of the studies’ methodological quality [19].

### Statistical analysis

After conducting an extensive review and extracting relevant data, studies that satisfied the inclusion criteria were integrated into a random effects diagnostic test accuracy (DTA) meta-analysis. The criteria for quantitative synthesis mandated the inclusion of true positive, true negative, false positive, and false negative values derived from diagnostic accuracy metrics reported in internal validation sets, including those utilizing n-fold cross-validation. For studies presenting multiple models, the primary model for each imaging modality was selected for the main meta-analysis, while additional models were included in subgroup analyses as appropriate.

We had an a priori assumption that diagnostic indices might differ among studies utilizing various imaging techniques. As a result, subgroup analyses were performed to compare these techniques within the meta-analysis. It was suggested that there might be significant differences in model performance based on their architecture, specifically between models employing deep learning feature extraction algorithms and those using HCR feature extraction methods. Additionally, we hypothesized that models based on radiomics or deep learning features from tumor ROIs versus those based on features from lymph node ROIs might influence performance. Thus, these factors were identified as critical variables for subgroup analyses.

The DTA meta-analysis employed the bivariate model proposed by Reitsma et al. [20]. Meta-regression using this model facilitated the exploration of differences between subgroups. Summary Receiver Operating Characteristic (SROC) curves were generated from the bivariate meta-analysis data, with study-specific estimates on these curves weighted according to their contributions within a random effects univariate Diagnostic Odds Ratio (DOR) model. To evaluate the overall diagnostic performance of the models, the area under the SROC curve (AUC) and its confidence intervals were calculated for each subgroup using 2000 sample bootstraps based on the bivariate model [21].

Heterogeneity was assessed using the I2 metric, based on the approach by Holling et al. [22]. An I2 confidence interval exceeding 25% indicated heterogeneity, prompting sensitivity analyses based on the DOR univariate meta-analysis to identify potential outliers. Identified outliers were re-analyzed to confirm the robustness of the findings. Additionally, publication bias was evaluated using a generalized Egger’s regression test for DTA meta-analysis, which examined funnel plot asymmetry using 2000 sample bootstraps, as recommended by Noma et al. [23].

All statistical analyses were conducted using the R software environment (version 4.2.1, R Foundation for Statistical Computing, Vienna, Austria), utilizing the R packages “Mada,” “MVPBT” [24], “dmetatools” [21], “Metafor” [25], and “meta” [26].

## Results

### Screening and selection of articles

**Fig. 1:**
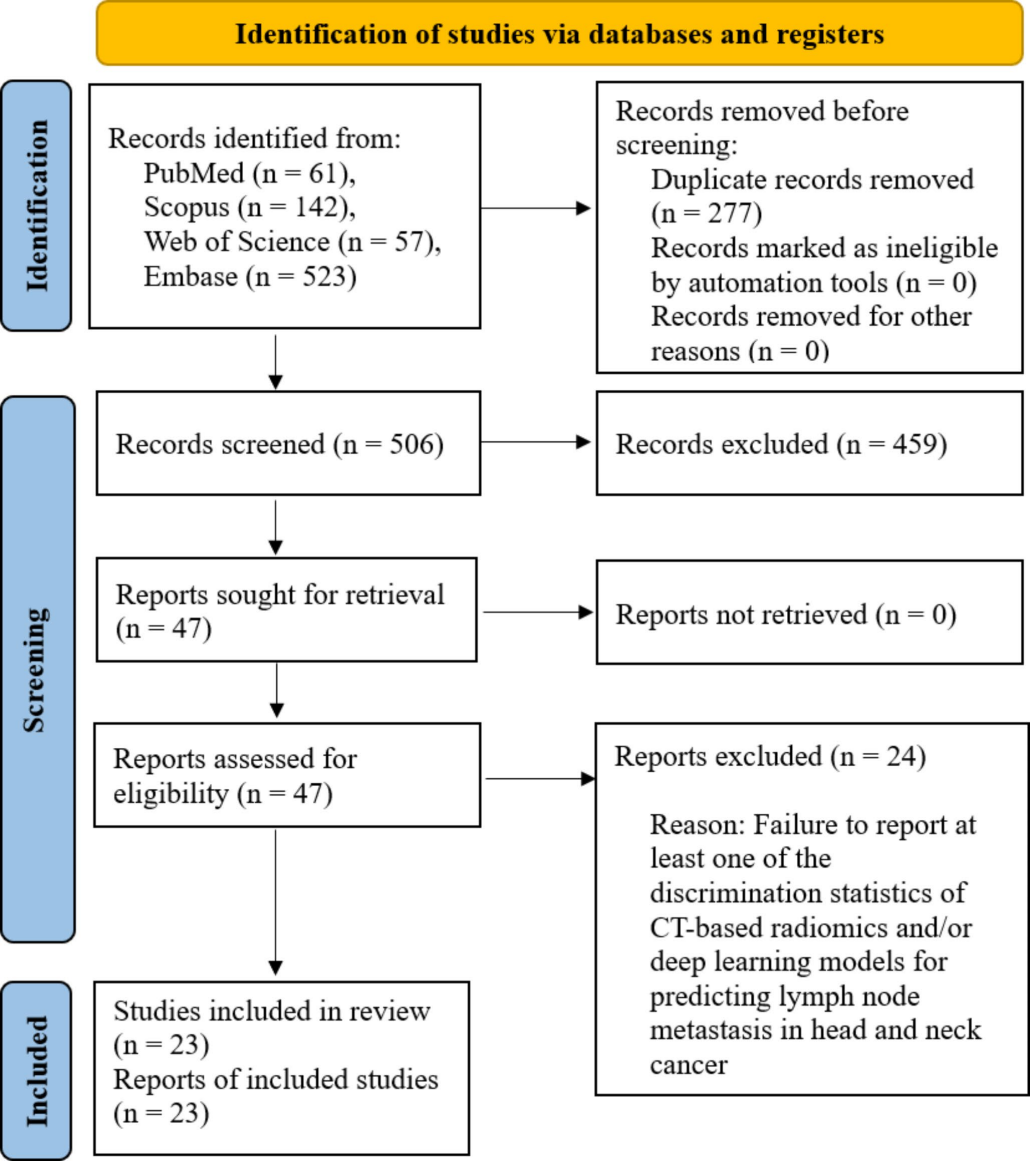
PRISMA flow diagram showing the review process, PRISMA: Preferred Reporting Items for Systematic Reviews and Meta-Analyses

A systematic literature search, following a predefined strategy, identified a total of 783 articles. After removing duplicates, 506 papers underwent initial screening based on the title and abstract, resulting in the exclusion of 459 articles. The full text of the remaining 47 papers underwent full-text review. Following a comprehensive examination, 24 articles were excluded as they did not align with the study’s aim. In the end, 23 articles that met the inclusion criteria were identified and included. The screening process and eligibility criteria adhered to PRISMA guidelines, and the PRISMA flow diagram illustrating the process is presented in Fig. 1.

## Study and patient characteristics

Twenty-three studies developing radiomics and/or deep learning models for diagnosing or predicting LNM in head and neck cancer patients were included in the study. Table 1 provides detailed demographic information from the chosen studies, describes the technical features of the models, and provides the diagnostic accuracy metrics for the primary models established by each study.

**Table 1:** Characteristics of the included studies and the models they developed

Author, Year	Imaging modality	Study sample	Assessed condition	Sample size	Gender (Female %)	Age	Reference test	Segmentation method	Specs of developed models	Specs of the main proposed model	Diagnostic performance of the main model (internal validation)	
Kudoh, 2023 [41]	PET	Patients with tongue SCC (first examination)	LNM during follow-up	40	32.50%	Mean ± SD: 66 ± 14	Clinical follow-up (± histopathology)	Semi-automatic 3D segmentation of regions with SUV of 2.5 or more followed by manual deletion of physiological uptake	Logistic regression models based on HCR features of the tumor in initial evaluation with different peritumoral margins and bins	Logistic regression model based on HCR features of the tumor in initial evaluation (3-mm; 10 bin)	Sens: 65% Spec: 70% AUC: 79% [69 − 89%]	
Ariji, 2019 [17]	CECT	Patients with oral SCC	LNM	41 (441 LNs)	46.67%	NS	Histopathology	Manual 2D rectangular ROIs	CNN based on DLR features to assess cervical LNM using LN images		Sens: 75% Spec: 81% AUC: 80%	
Ariji, 2019 [42]	Intra-oral doppler US	Patients with tongue SCC smaller than 4 cm (greatest dimension) with no cervical LNM involvement at first examination undergoing surgical tumor excision.	LNM during 2-year follow-up after operation (Partial glossectomy)	32 (134 images)	21.20%	NS	Histopathology	Manual 2D rectangular ROIs	CNN based on DLR features of the tumor		Sens: 84% Spec: 87% AUC: 88%	
Kann, 2018 [43]	CECT	Patients with non-metastatic HNSCC or salivary gland carcinoma undergoing cervical LN dissection	LN involvement in cervical lymph node dissection within 3 months after imaging	270 (653 LNs)	NS	NS	Histopathology	Manual 3D segmentation of the LN	CNNs based on DLR features and a random forest model based on HCR to diagnose LNM with and without clinical features	CNN (DualNet) based on DLR features of the LN	Sens: 84% Spec: 87% AUC: 88%	
Chen, 2019 [34]	CT, PET-CT, PET	Patients with head and neck carcinoma	LN involvement or “suspicious” LN involvement	41 (170 LN)	NS	NS	Consensus assessment by a radiation oncologist and a nuclear medicine radiologist based on the images and clinical status	Manual 3D segmentation of the LN	Models with CNN and SVM classifier with HCR and/or DLR features from different modalities (CT, PET-CT, PET)	Hybrid model (CNN model based on HCR and DLR features of the LN)	PET: Sens: 98% Spec: 82%	
											PET-CT: Sens: 94% Spec: 94%	
											CT: Sens: 98% Spec: 82%	
Chen, 2021 [16]	PET-CT	Patients with oropharyngeal SCC undergoing neck LN dissection and with preoperative PET + CE-CT	LN involvement	129 (791 LNs)	NS	NS	Histopathology	Manual 3D segmentation of the LN	Models with CNN classifiers with and without attention guiding and a model with SVM classifiers based on HCR features of the LN.	Attention-guided CNN model based on DLR features of the LN	Sens: 91% Spec: 93% AUC: 98%	
Wang, 2021 [44]	MRI	Patients with tongue cancer treated by neck LN dissection and preoperative MRI within 30 days	LN involvement	236	39%	50.8 (± 13.6)	Histopathology	Manual 3D segmentation of the tumor	Models with SVM classifiers based on HCR features with and without clinical features with tumoral ROIs with different levels of peritumor area	SVM model based on HCR features of the tumor and 10 mm peritumoral area in MRI imaging (T2) along with clinical features	Sens: 79% Spec: 93% AUC: 87%[84 − 89%]	
Wang, 2022 [45]	MRI	Patients with HNSCC treated by neck LN dissection with preoperative MRI	LN involvement	160	26%	55.6 (± 14.4)	Histopathology	Manual 3D segmentation of the LN	Models with LR classifier based on HCR with and without ADC values and maximum diameter of the LN.	LR model based on HCR features of the LN in DWI and CE-T1 imaging, along with data on max diameter and raw ADC values	Sens: 83% Spec: 76% AUC: 83%	
Tomita, 2021 (a) [46]	CECT	Patients with oral SCC treated by neck LN dissection with preoperative CE-CT	LN involvement	23 (201 LN)	43%	52 (± 8)	Histopathology	Manual 2D segmentation of the LN	Models with SVM classifier based on HCR features from LN and features such as short-diameter	SVM model based on HCR features of the LN, along with short diameter value	Sens: 80% Spec: 100% AUC: 93% [86 − 96%]	
Tomita, 2021 (b) [47]	CECT	Patients with oral SCC undergoing neck LN dissection with preoperative CT	LN involvement	39 (320 LN)	41%	64 (± 14)	Histopathology	Manual 2D segmentation of the LN	A model with CNN classifier based on DLR features of LNs		Sens: 67% Spec: 95% AUC: 90% [78 − 96%]	
Ren, 2022 [48]	MRI	Oral tongue SCC	Occult LN involvement	55	44%	53 (± 10.2)	Histopathology	Manual 3D segmentation of the tumor	A model with LR classifier based on HCR features of wash-in and washout DCE-MRI and ADC and MRI-based depth of invasion of the tumor		Sens: 79% Spec: 86% AUC: 87% [77 − 96%]	
Seidler, 2019 [49]	DECT	HNSCC patients with neck dissection pathological specimen	LN involvement	50 (412 LN)	50%	69	Histopathology	Manual 2D segmentation of the LN	Models with GBM and RF classifiers based on HCR features of LNs in DE-CT imaging	Models with GBM classifier based on HCR features of LNs in DE-CT imaging	Sens: 100% Spec: 86% AUC: 96% [87 − 100%]	
Xu, 2023 [28]	CECT	Oral cancer patients treated by elective LN dissection with preoperative CT	LN involvement	1466 (5601 LN)	28%	(median [IQR]) 55.41 [48, 64]	Histopathology	Automatic segmentation of neck LNs	Models with CNN architecture based on DLR features of LNs, using auto-segmented LN features from a segmentation model. With and without transfer learning.	CNN based on CE-CT images with transfer learning	Sens: 70.4% Spec: 73%	
Dohopolski, 2020 [50]	PET-CT	Oropharyngeal SCC patients undergoing neck dissection with preoperative PET-CT	LN involvement	129 (791 LN)	NS	NS	Histopathology	Manual 2D rectangular	CNN model based on DLR features of PET-CT images of the LN		Sens: 94% Spec: 90% AUC: 99%	
Lu, 2022 [15]	MRI	Hypopharyngeal SCC patients treated by neck LN dissection with tumor larger than 1 cm (largest dimension)	LN involvement	155	NS	58.9 (± 9.3)	Histopathology	Manual 3D	Models with LR classifiers based on HCR features of the LN, with and without clinical features	LR model based on HCR features of the LN with clinical data	AUC: 85% [74 − 97%]	
Konishi, 2023 [51]	Intra-oral US	Tongue cancer with negative node	late cervical LNM	120	42%	62.2 (± 17.1)	Histopathology	Manual 2D	Models with BF, SVM, and NTB classifiers based on HCR features from primary tumor	Model with NTB classifier based on HCR features from the primary tumor	AUC: 97%	
Ho, 2020 [52]	MRI	HNSCC patients with LN histopathology results and preoperative MRI	LN involvement	25 (68 LN)	NS	NS	Histopathology	Manual 3D segmentation of LN	Models with multilayer perception neural network classifiers based on HCR features of LN, with and without feature selection	Model with multilayer perceptron neural network classifiers based on HCR features of LN, with feature selection	Sens: 79% Spec: 69%	
Forghani, 2019 [14]	DECT	HNSCC patients with DE-CT imaging	Nodal status	87	37%	68 (range: 43–96)	Histopathology	Manual 2D	Model with RF classifier based on HCR features of DECT of the tumor		Sens: 100% Spec: 67%	
Committeri, 2022 [53]	CECT	Early-stage OTSCC patients with CE-CT imaging and at least 12 months of follow-up	LN involvement	81	60.50%	median: 58 (range: 19–86)	Clinical follow-up (± histopathology)	Manual 3D	Models with LR and decision forest-based classifiers based on HCR features of the LN, with and without clinical data.	Decision forest-based model with HCR features of the LN (clinical data was present in the model but was not used for decision-making)	Sens: 100% Spec: 100%	
Zhong, 2022 [54]	CECT	Patients with tongue SCC and enlarged cervical LN undergoing primary tumor resection and neck dissection with CE-CT less than 20 days before surgery	Nodal status	313	40%	55.1 (± 12.4)	Histopathology	Manual 3D	Models with ANN classifiers based on HCR features from the tumor, with different levels of hyperparameters	ANN based on HCR features of the tumor	Sens: 93.1% Spec: 76.5% AUC: 94.3% [89.1 − 99.6%]	
Zhao, 2023 [55]	CECT	Laryngeal SCC patients undergoing open surgery and lymphadenectomy	LN involvement	464	5%	62 (± 8.8)	Histopathology	Manual 3D	Model with LR classifier based on HCR features of the tumor and clinical features, including CT reports of the LN		Sens: 86.1% Spec: 84.1% AUC: 91%	
Yuan, 2021 [29]	MRI	OTSCC patients undergoing primary tumor excision and neck LN dissection	LN involvement	116	41%	55 (± 12)	Histopathology	Manual 3D	Models with different classifiers based on HCR features of the tumor in T2W and/or CE-T1 images	NB model with HCR features from CE-1 W and T2W images of the tumor	Sens: 63.3% Spec: 82.1% AUC: 80.2%	
Kubo, 2022 [27]	CECT	Patients with tongue cancer undergoing primary excision and no LNM before treatment	OCLNM based on 1 year clinical follow-u	161	50.30%	median: 65, (range: 22–91)	Clinical follow-up (± histopathology)	Manual 3D	Models with different classifiers based on HCR features of single LN or whole neck level LNs, with and without using SMOTE resampling methods.	SVM model based on HCR features of each neck node level with SMOTE resampling	Sens: 66% Spec: 85% AUC: 98%	

**Abbreviations**: OCLNM: occult cervical lymph node metastasis; 2D: Two dimensional; 3D: Three dimensional; ADC: apparent diffusion coefficient; ANN: artificial neural network; AUC: area under the curve; BF: Bootstrap forest; CECT: contrast-enhanced computed tomography; CNN: convolutional neural network; DCE-MRI: dynamic contrast-enhanced magnetic resonance imaging; DECT: Dual-energy computed tomography; DLR: deep learning-based radiomics; DualNet: dual network; DWI: Diffusion weighted imaging; GBM: Gradient Boosting Machine; HCR: Handcrafted radiomics; HNSCC: Head and neck squamous cell carcinoma; IQR: interquartile range; LN: lymph node; LNM: lymph node metastasis; LR: logistic regression; MRI: magnetic resonance imaging; NB: Naive Bayes; NS: not specified; NTB: Neural tanh boost; OTSCC: oral tongue squamous cell carcinoma; PET: positron emission tomography; RF: Random Forest; ROI: region of interest; SCC: Squamous cell carcinoma; SD: standard deviation; Sens: Sensitivity; SMOTE: Synthetic Minority Over-sampling Technique; Spec: Specificity; SUV: standardized uptake value; SVM: Support vector machines; T2W = T2-weighted; US: ultrasoud

The included studies developed models using features extracted from various imaging modalities. CT imaging was used in 12 studies, MRI in six, PET/CT in three, while PET and intraoral US were each used in two studies. Furthermore, in most studies, the reference standard for evaluation was the surgical histopathology of lymph nodes obtained from dissection. Fourteen studies focused on analyzing radiomics features derived from lymph nodes, whereas nine studies focused on the features of primary tumors. In terms of the radiomics features utilized, 18 studies used hand-crafted features to develop their models, whereas seven studies integrated deep learning algorithms for feature extraction. Of these, two studies combined deep learning with HCR features, and five exclusively employed deep learning features.

## Quality assessment

The quality of the models in the included studies was assessed using the METRICS checklist, with the comprehensive results displayed in **Table A.1**. This analysis showed a median METRICS score of 73.3%. Scores varied from a minimum of 58.1% to a maximum of 95.2%, revealing “good” methodological quality in most of the included studies, with certain concerns especially regarding the lack of validation, robustness, and generalizability of the models. Among the included studies, only one had an external validation set [43], while many lacked appropriate internal or external validation methods, which are critical for preventing information leakage.

### Meta-analysis of models based on CT, MRI, and PET/CT

**Fig. 2:**
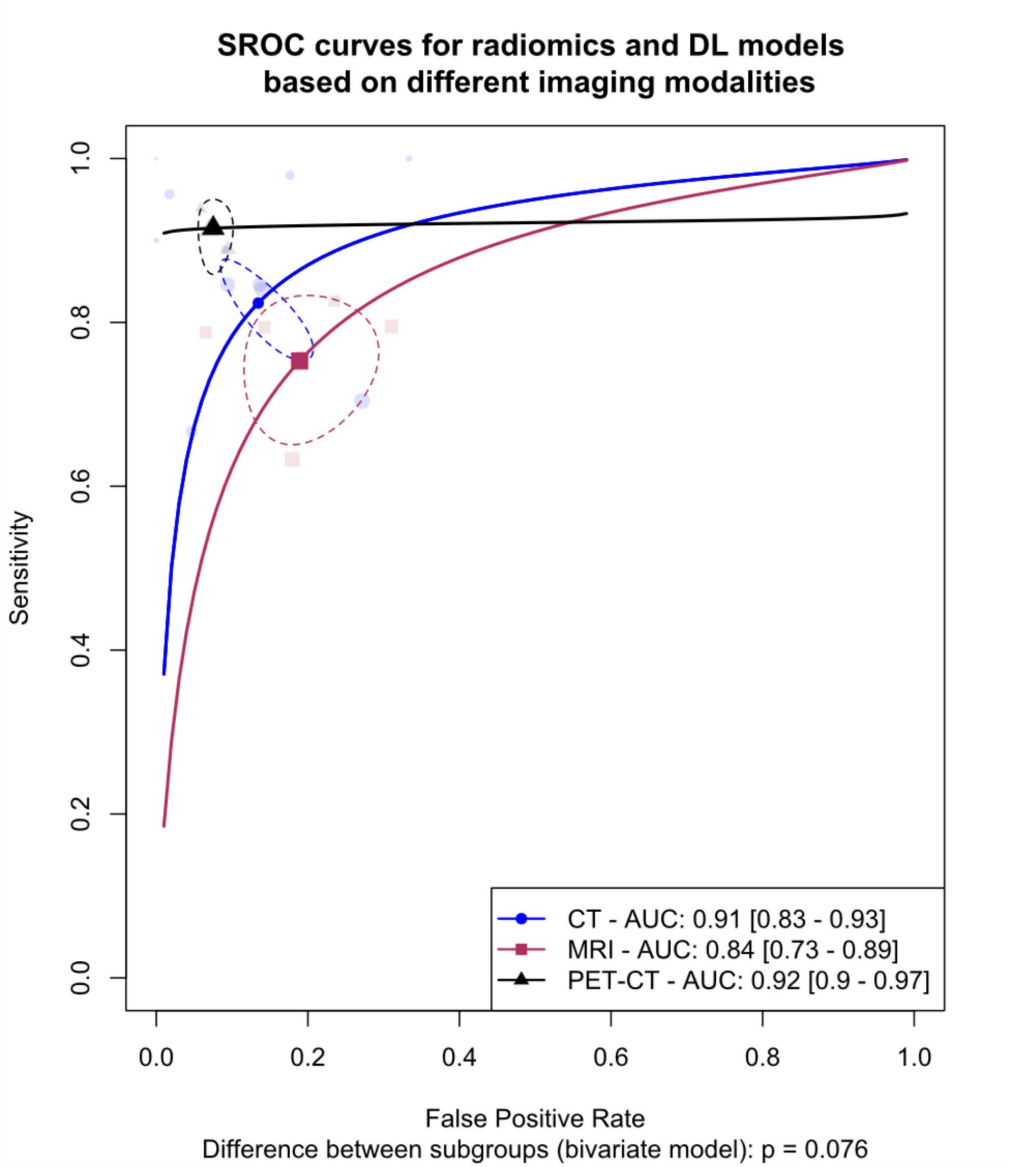
Summary Receiver Operating Curves (SROCs) for subgroup meta-analysis comparing models based on different modalities, The between-group difference is derived from the bivariate model, AUC: Area under the curve. DL: Deep learning

We evaluated the differences in diagnostic accuracy among models using features derived from CT, MRI, and PET/CT. Due to the absence of external validation in most studies, all analyses were confined to internal validation sets. Figure 2 demonstrates the SROC curves comparing the diagnostic accuracy across these imaging modalities. Our findings indicated that the pooled AUC values were 91% (95% CI: 83-93%) for CT-based models, 84% (95% CI: 73-89%) for MRI-based models, and 92% (95% CI: 90-97%) for PET/CT-based models. This analysis suggests a trend towards a difference in diagnostic accuracy among these modalities (*p* = 0.076).

**Fig. 3:**
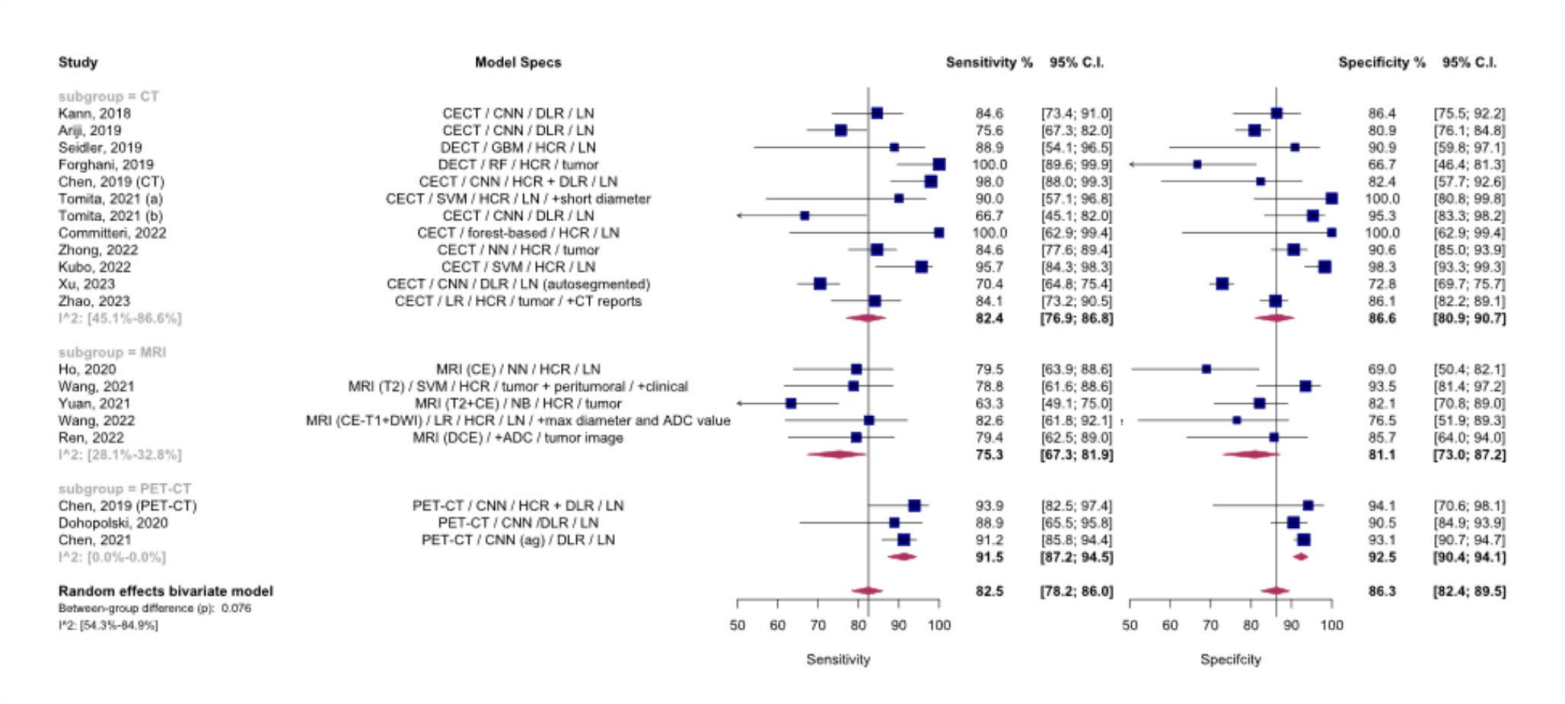
Paired Forest plots for the subgroup meta-analysis comparing models based on different modalities, The between-group difference is derived from the bivariate model, ADC: apparent diffusion coefficient. ag: attention-guided. CE: contrast-enhanced. CECT: contrast-enhanced CT. CI: Confidence interval. CNN: Convolutional neural network. DCE: dynamic contrast-enhanced. DECT: dual-energy CT. DLR: Deep learning radiomics. DWI: diffusion-weighted imaging. GBM: Gradient Boosting Machine. HCR: hand-crafted radiomics. LN: lymph node. LR: logistic regression. NB: naïve Bayes. NN: neural network. RF: Random forest. SVM: Support vector machine

Paired forest plots for this analysis are represented in Fig. 3, showing pooled sensitivity and specificity of 82.4% (95% CI: 76.9-86.8%) and 86.6% (95% CI: 80.9-90.7%) for CT, 75.3% (95% CI: 67.3-81.9%) and 81.1% (95% CI: 73.0-87.2%) for MRI, and 91.5% (95% CI: 87.2-94.5%) and 92.5% (95% CI: 90.4-94.1%) for PET/CT, respectively. Due to the marginally significant difference observed in the bivariate model, a post-hoc analysis was performed, indicating a higher sensitivity for PET/CT-based models (*p* = 0.02).

Substantial heterogeneity was observed within CT (I^2^: 45.1 − 86.6%) and MRI (I^2^: 28.1 − 32.8%) subgroups. The leave-one-out analysis identified studies by Kubo et al. (2022) and Xu et al. (2023) as outliers in the CT subgroup [27, 28]. Following their exclusion, **Fig. A.1** shows a revised forest plot, and the statistical significance of differences in diagnostic accuracy among the imaging modalities was confirmed (*p* < 0.01). Further post-hoc analyses also revealed higher sensitivity and specificity for PET/CT models (*p* < 0.01) after excluding these outlier studies.

## Meta-analysis of deep learning versus hand-crafted radiomics models

**Fig. 4:**
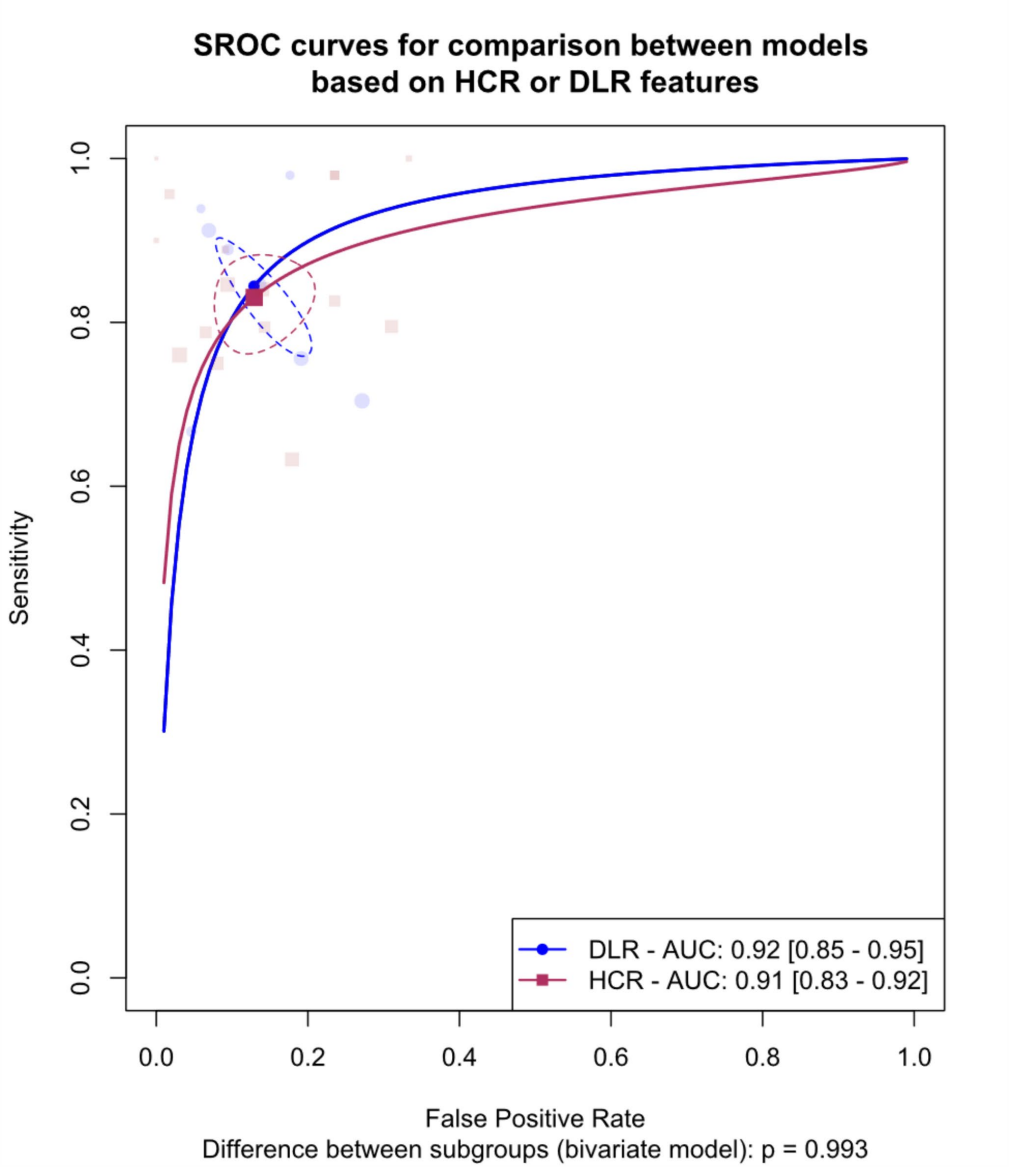
Summary Receiver Operating Curves (SROCs) for subgroup meta-analysis comparing models based on deep learning vs. hand-crafted radiomics, The between-group difference is derived from the bivariate model, AUC: Area under the curve. DLR: Deep learning radiomics. HCR: hand-crafted radiomics

We explored the differences between models that utilize deep learning algorithms for feature extraction and those that employ HCR features. The analysis was limited to internal validation sets due to the lack of external validation in most of the included studies. Figure 4 displays the SROC curves for these model types. The pooled AUC was 92% (95% CI: 85-95%) and 91% (95% CI: 83-92%) for deep learning models and HCR models, respectively, with no significant difference in diagnostic accuracy observed (*p* = 0.993).

**Fig. 5:**
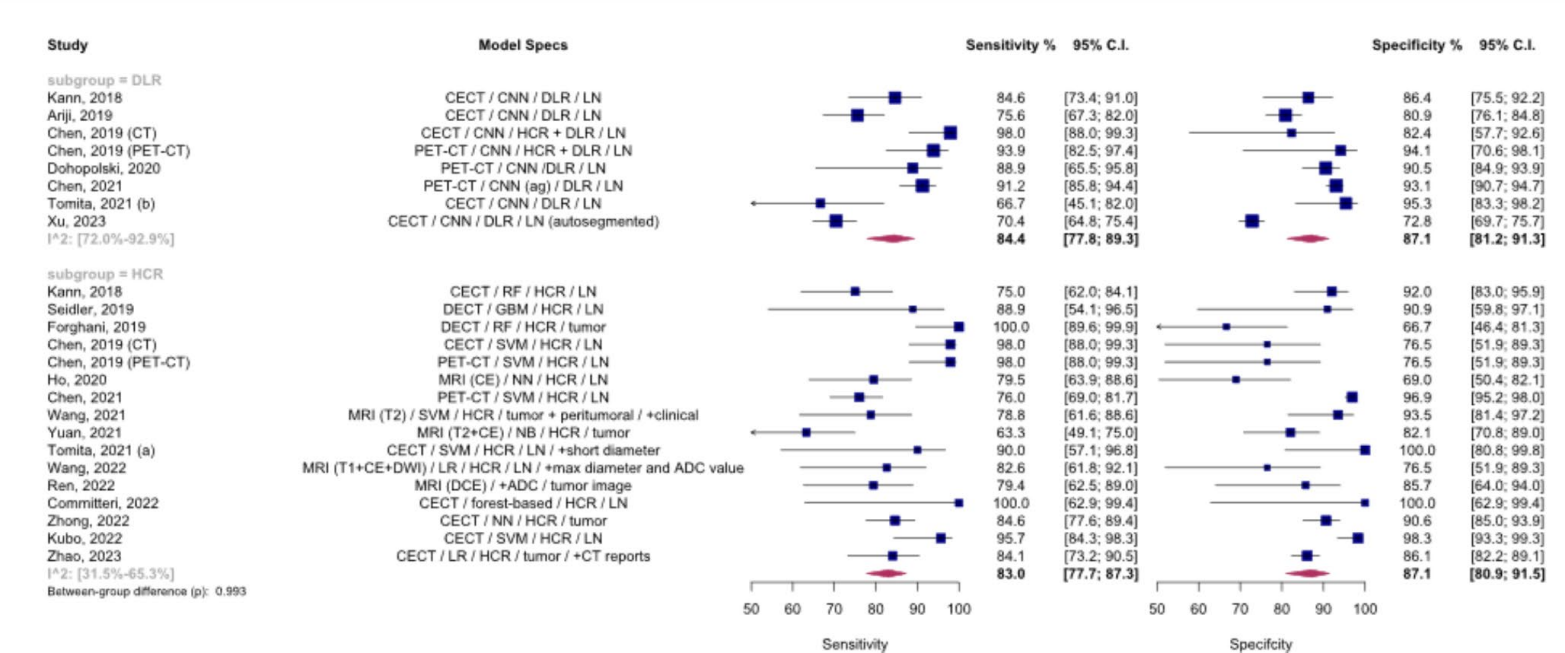
Paired forest plots for the subgroup meta-analysis comparing models based on deep learning vs. hand-crafted radiomics, The between-group difference is derived from the bivariate model, ADC: apparent diffusion coefficient. ag: attention-guided. CE: contrast-enhanced. CECT: contrast-enhanced CT. CI: Confidence interval. CNN: Convolutional neural network. DCE: dynamic contrast-enhanced. DECT: dual-energy CT. DLR: Deep learning radiomics. DWI: diffusion-weighted imaging. GBM: Gradient Boosting Machine. HCR: hand-crafted radiomics. LN: lymph node. LR: logistic regression. NB: naïve Bayes. NN: neural network. RF: Random forest. SVM: Support vector machine

Figure 5 presents paired forest plots for this analysis. The corresponding sensitivities and specificities were 83.0% (95% CI: 77.7-87.3%) and 87.1% (95% CI: 80.9-91.5%) for HCR models, and 84.4% (95% CI: 77.8-89.3%) and 87.1% (95% CI: 81.2-91.3%) for deep learning models, respectively, with no significant difference (*p* = 0.993). Substantial heterogeneity was present in both groups (DL: I²: 72-92.9%, HCR: I²: 31.5-65.3%). The leave-one-out analysis identified Kubo et al. (2022) and Yuan et al. (2021) as outliers in the HCR group [27, 29], and the forest plot excluding these studies is presented in **Fig. A.2**. After excluding the outliers, the difference in diagnostic accuracy remained insignificant (*p* = 0.982).

## Meta-analysis of models based on radiomics features from lymph nodes versus primary tumor

**Fig. 6:**
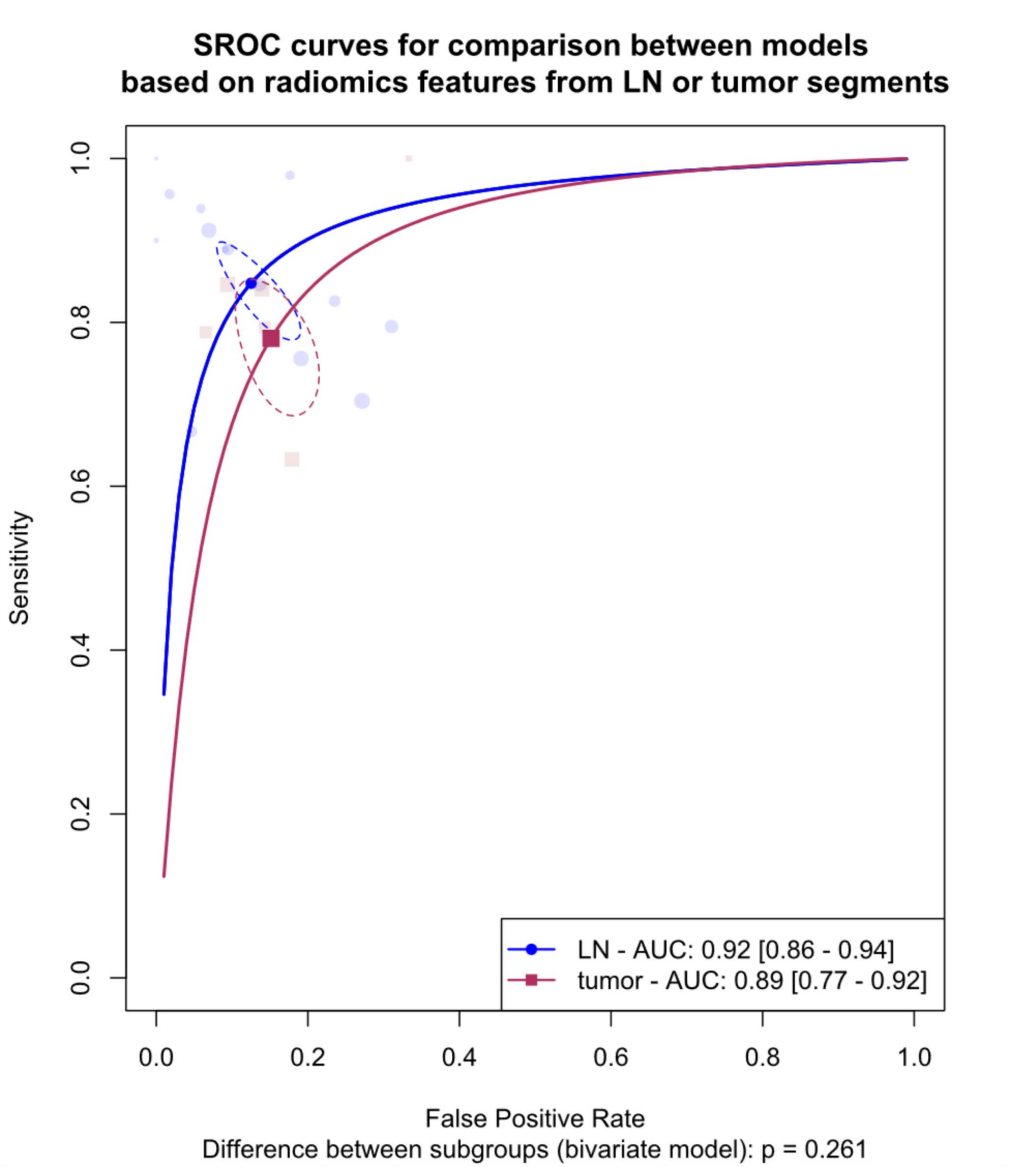
Summary Receiver Operating Curves (SROCs) for subgroup meta-analysis comparing models based on radiomics features extracted from lymph nodes vs. primary tumor, The between-group difference is derived from the bivariate model, AUC: Area under the curve. LN: lymph node

We explored differences between models based on radiomics features from lymph nodes versus primary tumors. The analysis was limited to internal validation sets due to the lack of external validation in most of the included studies. Figure 6 shows the SROC curves comparing the two groups, revealing a pooled AUC of 92% (95% CI: 86-94%) for lymph node models versus 89% (95% CI: 77-92%) for primary tumor models, with no significant difference observed (*p* = 0.261).

**Fig. 7:**
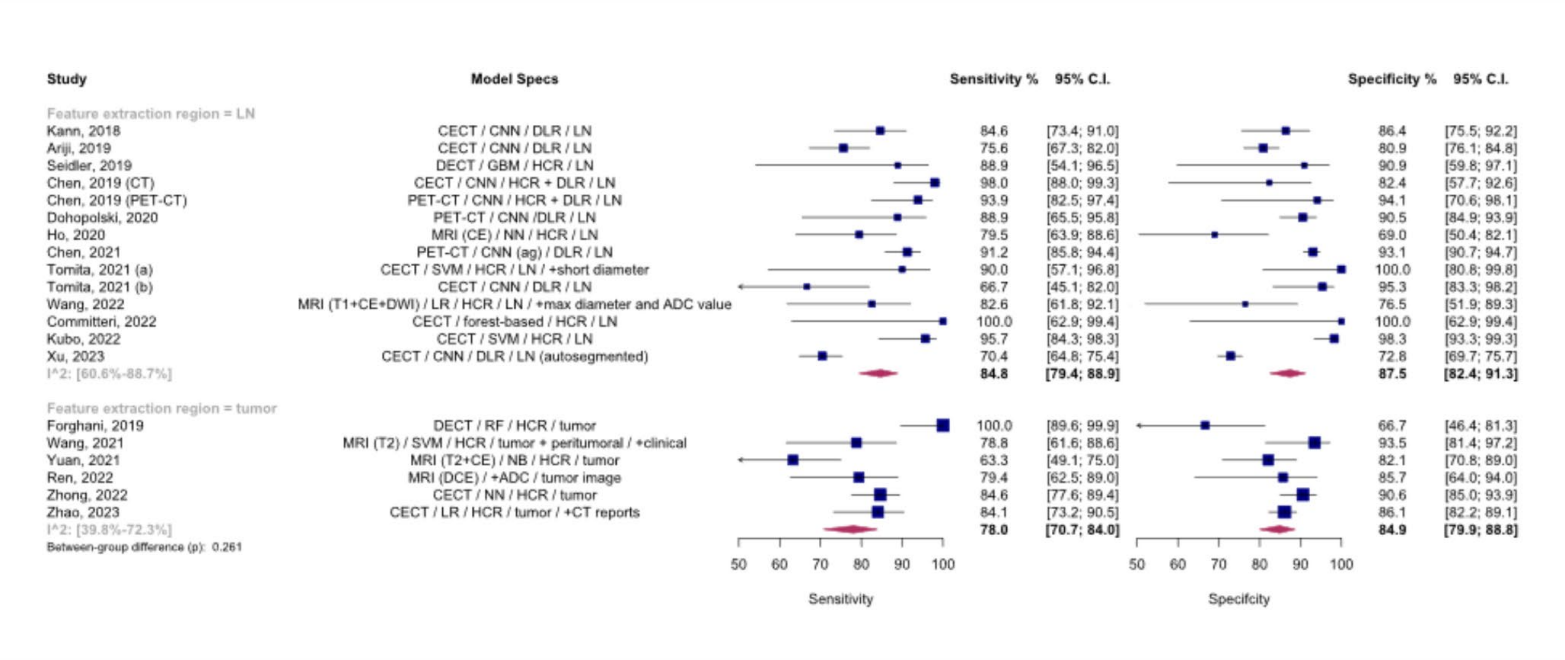
Paired forest plots for the subgroup meta-analysis comparing models based on radiomics features extracted from lymph nodes vs. primary tumor. ADC: apparent diffusion coefficient. ag: attention-guided, CE: contrast-enhanced. CECT: contrast-enhanced CT. CI: Confidence interval. CNN: Convolutional neural network. DCE: dynamic contrast-enhanced. DECT: dual-energy CT. DLR: Deep learning radiomics. DWI: diffusion-weighted imaging. GBM: Gradient Boosting Machine. HCR: hand-crafted radiomics. LN: lymph node. LR: logistic regression. NB: naïve Bayes. NN: neural network. RF: Random forest. SVM: Support vector machine. The between-group difference is derived from the bivariate model

Figure 7 presents paired forest plots for this analysis, demonstrating pooled sensitivity and specificity of 84.8% (95% CI: 79.4-88.9%) and 87.5% (95% CI: 82.4-91.3%) for lymph node models and 78.0% (95% CI: 70.7-84.0%) and 84.9% (95% CI: 79.9-88.8%) for primary tumor models, respectively.

Substantial heterogeneity was observed within both lymph node (I2: 60.6 − 88.7%) and primary tumor (I2: 39.8 − 72.3%) groups. Leave-one-out analysis identified the study by Yuan et al. (2021) as a significant outlier in the primary tumor group [29]. The absence of a significant difference was consistent after the exclusion of this study, as shown in **Fig. A.3** (*p* = 0.736).

### Assessment of publication bias

**Fig. 8:**
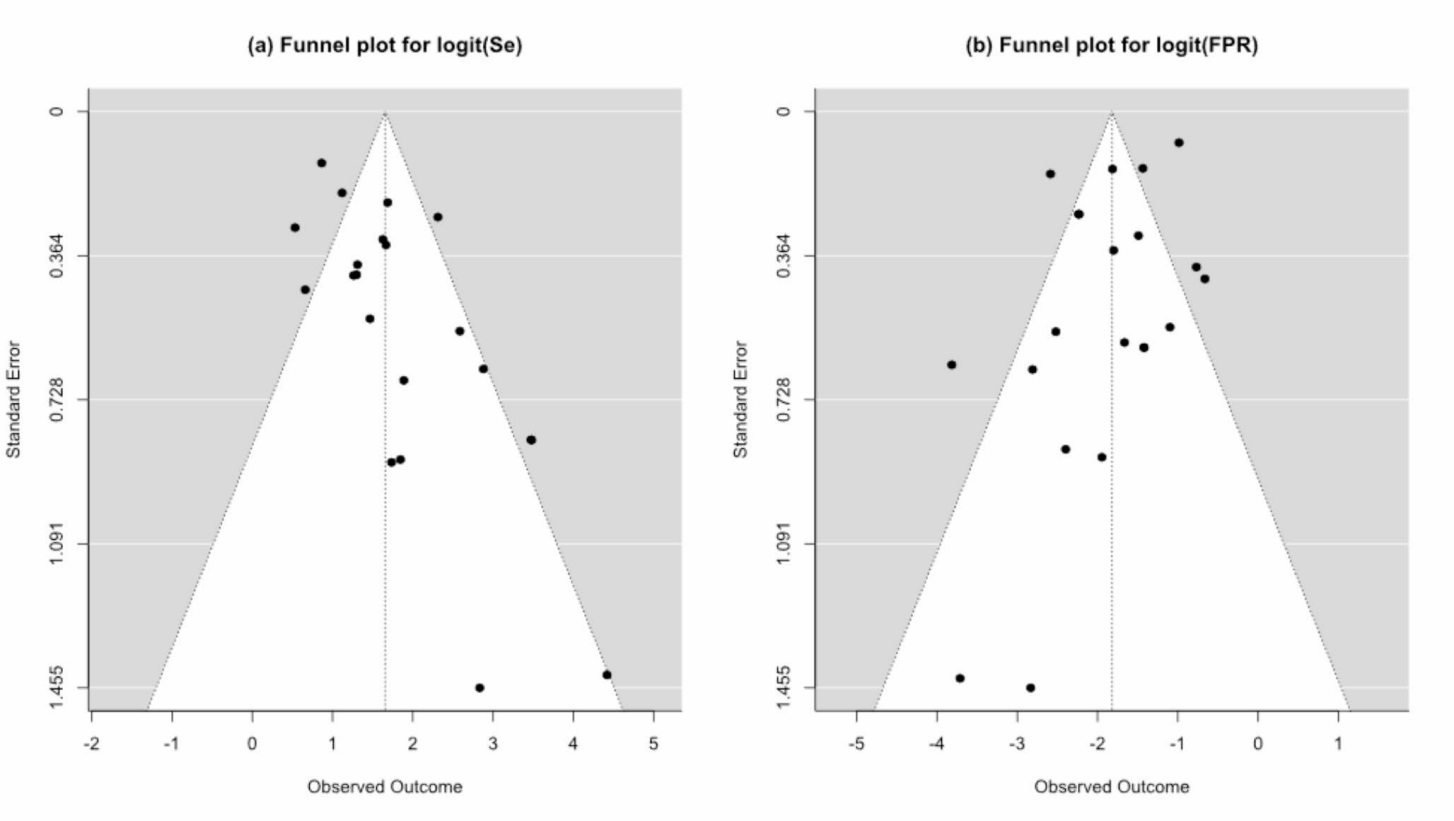
Paired funnel plots are used to assess potential publication bias/small study effect among reported values for diagnostic accuracy of the main models of each study, FPR: False positive rate, Se: Sensitivity

Figure 8 depicts paired funnel plots used to assess publication bias and small study effects in the diagnostic accuracy reported by the primary models of each study. A significant publication bias was confirmed through Generalized Egger’s regression test (*p* < 0.005).

## Discussion

The result of the present systematic review and meta-analysis demonstrates the promising accuracy of radiomics and deep learning models in diagnosing LNM in head and neck cancers, with a pooled AUC of 91%, 84%, and 92% for CT-based models, MRI-based models, and PET/CT-based models, respectively. We also retrieved a pooled AUC of 92% and 91% for deep learning and HCR models, respectively. These models showed acceptable accuracy across different imaging modalities, including CT, MRI, and PET/CT, as well as different pipelines, including those based on cervical LN images and those based on tumor images, shedding light on the potential clinical application of such models in clinical practice.

Our findings are in line with several meta-analysis studies showing the promising capabilities of precision medicine and radiomics pipelines in diagnosing and predicting LNM in various types of cancers, including breast cancer [30], biliary tract malignancies [31], and colorectal cancer [32]. For instance, the review by Windsor et al. discussed breast cancer LNM prediction using radiomics models based on different modalities and reported excellent pooled diagnostic accuracy metrics of Artificial Intelligence (AI)-based models in LNM prediction across various imaging modalities [30]. Notably, an included study in their review [33] reported improved sensitivity of radiologists’ LMN detection while working collaboratively with AI models, highlighting the importance of integrating radiomics pipelines in the clinical practice of radiologists, leveraging both visual assessment of radiologists and the radiomics features assessed by machine learning algorithms.

Our meta-analysis embodied three subgroup analyses, in which the included studies were categorized based on the method they utilized to extract features (deep learning vs. HCR), their ROIs (the lymph nodes vs. the primary tumor), and their imaging modality (CT vs. MRI vs. PET/CT). the only analysis where a statistically significant difference was observed between the subgroups was the latter, with PET/CT consistently showing higher accuracy both before and after the exclusion of the outlier studies.

Aside from the higher image quality and the more detailed training data with PET/CT, another attributing factor to this finding could be explored within the nuances of the deployed AI model, such as the use of attention-guided classification (AGC) in one of the three PET/CT studies that were included, which was also the one with the highest obtained sensitivity and specificity. AGC consists of 2 modules: (1) an attention-guided convolutional neural network (agCNN) and (2) a classification CNN (cCNN) [16]. Chen et al. reported that their AGC model outperformed both conventional CNNs and radiomics models. What sets AGC apart from conventional CNNs is the incorporation of human knowledge into the training process as well as the unnecessity of accurate delineation [16]. This will enable the agCNN module to identify useful regions within the ROI patch and feed it to the classification CNN, hence the enhanced accuracy.

In another PET/CT study, Chen et al. developed a hybrid model using a many-object radiomics (MaO-radiomics) and a 3D-CNN and fused their outputs using an evidential reasoning approach [34]. The study suggests that utilizing a radiomics model alongside a deep learning model could help experts leverage the advantages of both models and optimize accuracy.

One of the main advantages of AI-based models is the promise of early and accurate prediction of LNM, which can bring about a paradigm shift in cancer management and significantly improve patients’ outcomes [35]. Also, the development and utilization of non-invasive methods for LNM detection will obviate the need to impose costly, time-consuming, and invasive procedures on the patients. More to the point, contemporary methods of detecting LNM in numerous cancerous conditions are highly prone to inter-observer variability, which can confound the robustness of the findings [35], while AI-based models, based on robust, reproducible, and explainable pipelines can potentially increase the confidence and accuracy and attain more robust results.

The use of radiomics models in medical imaging, however, has its fair share of challenges. For example, hardware limitations must be resolved to acquire high-quality data. Moreover, multidisciplinary teams must be formed to set standards and regulate confidentiality aspects in order to ensure the auspicious application of AI in clinical practice [36]. Another concerning obstacle to applying AI in medical imaging is overfitting. Overfitting occurs when the AI model is no longer generalizable to the whole population and only works on the training data [37–39]. In order to overcome overfitting, data augmentation and a higher sample size, particularly one that is a true representative of the whole population, can be helpful [37]. Finally, uncertainty quantification methods must be incorporated into AI-based medical imaging in order to improve accuracy and help radiologists additionally scrutinize highly uncertain predictions and confirm or reject them [16, 40]. Most current radiomics studies lack these methodological strengths, which poses a major concern regarding the clinical applicability of the developed models. Consequently, more effort is required to develop more reliable machine-learning methods for implementation in clinical practice.

There are a number of limitations to this study. First, the substantial amount of heterogeneity observed among the included studies limits the generalizability of the findings. Second, it was confirmed that a significant publication bias exists, which calls for a thorough revision in the process of reviewing, publishing, and interpreting AI research. Third, the included studies were primarily single-centric, lacking multi-centric external validation, which further limits the generalizability of the findings. Most of the included studies used k-fold cross-validation, introducing bias through the risk of overfitting and information leakage, and did not provide sufficient data to verify whether all appropriate measures were taken to prevent information leakage; furthermore, there were not enough studies to do a comprehensive subgroup analysis comparing studies with k-fold internal validation to the others. To the best of our knowledge, however, this is the first comprehensive systematic review and meta-analysis on the diagnostic accuracy of AI models in LNM detection in head and neck cancers.

## Conclusion

The great potential of AI in LNM prediction was ascertained in our meta-analysis. Deep learning and HCR models showed similarly excellent performance in detecting LNM in head and neck cancers, and PET/CT imaging turned out to be significantly associated with higher accuracy metrics. Nevertheless, further research is required to illuminate the clinical implications, pitfalls, and full potential of AI-based models in LNM detection.

## Electronic supplementary material

Below is the link to the electronic supplementary material.

Supplementary Material 1
