# Supplementary material for: Diagnostic accuracy of radiomics and artificial intelligence models in diagnosing lymph node metastasis in head and neck cancers: a systematic review and meta-analysis
Source: Neuroradiology. 2024 Nov 11;67(2):449–67. doi: 10.1007/s00234-024-03485-x (PMC11893643; doi:10.1007/s00234-024-03485-x)
Supplement: Supplementary file 1 — Supplementary Material 1 [file 234_2024_3485_MOESM1_ESM.docx]

**Article title:**

Diagnostic Accuracy of Radiomics and Artificial Intelligence (AI) Models in Diagnosing Lymph Node Metastasis in Head and Neck Cancers; A Systematic Review and Meta-Analysis

**Journal name:**

Neuroradiology

**Correspondence:**

Ali Gholamrezanezhad, MD

Department of Radiology, Keck School of Medicine, University of Southern California (USC), 1441 Eastlake Ave Ste 2315, Los Angeles, CA 90089, United States; Phone Number: +1 (443) 839-7134; Email: [Ali.Gholamrezanezhad@med.usc.edu](mailto:Ali.Gholamrezanezhad@med.usc.edu)


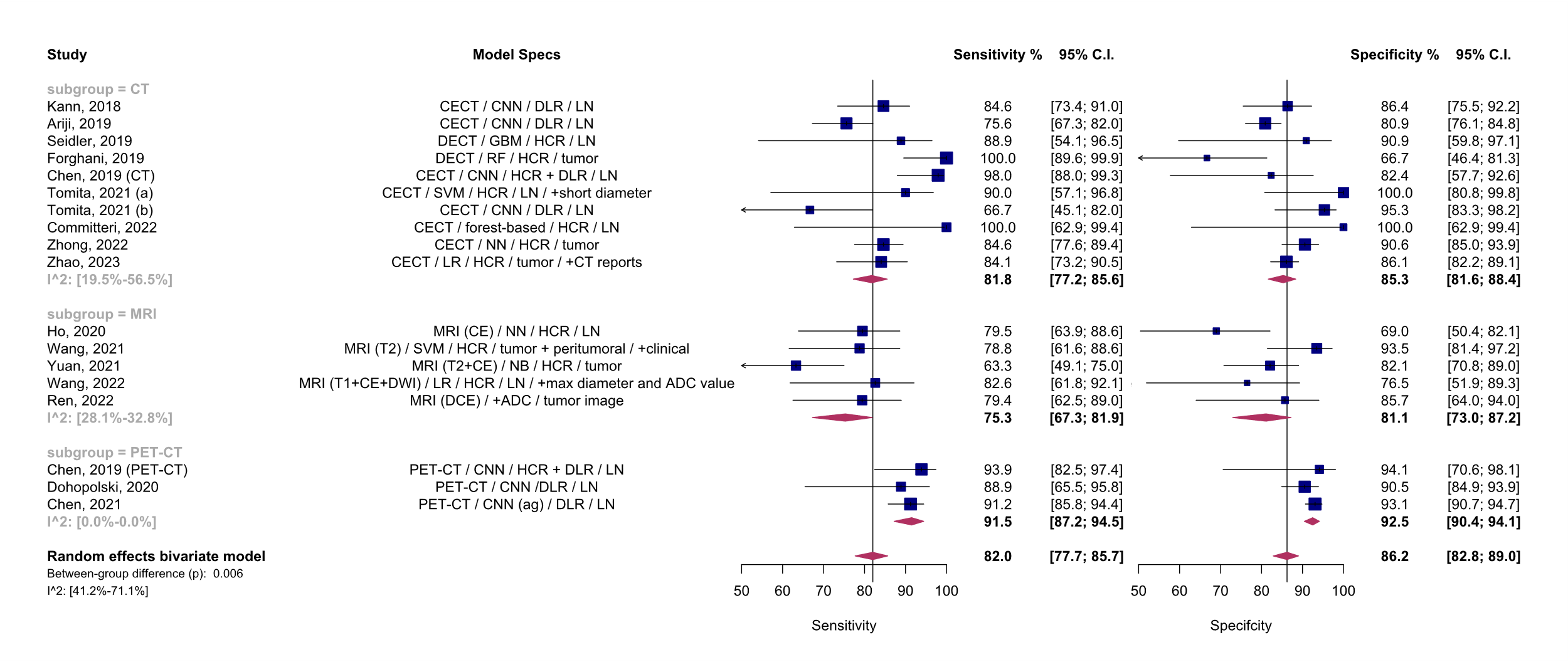


**Fig. A. 1** Paired Forest plots for the subgroup meta-analysis comparing models based on different modalities, after excluding outlier studies, The between group difference is derived from bivariate model, ADC: apparent diffusion coefficient. ag: attention-guided. CE: contrast-enhanced. CECT: contrast-enhanced CT. CI: Confidence interval. CNN: Convolutional neural network. DCE: dynamic contrast-enhanced. DECT: dual energy CT. DLR: Deep learning radiomics. DWI: diffusion weighted imaging. GBM: Gradient Boosting Machine. HCR: hand-crafted radiomics. LN: lymph Node. LR: logistic regression. NB: naïve baYes. NN: neural network. RF: Random forest. SVM: Support vector machine


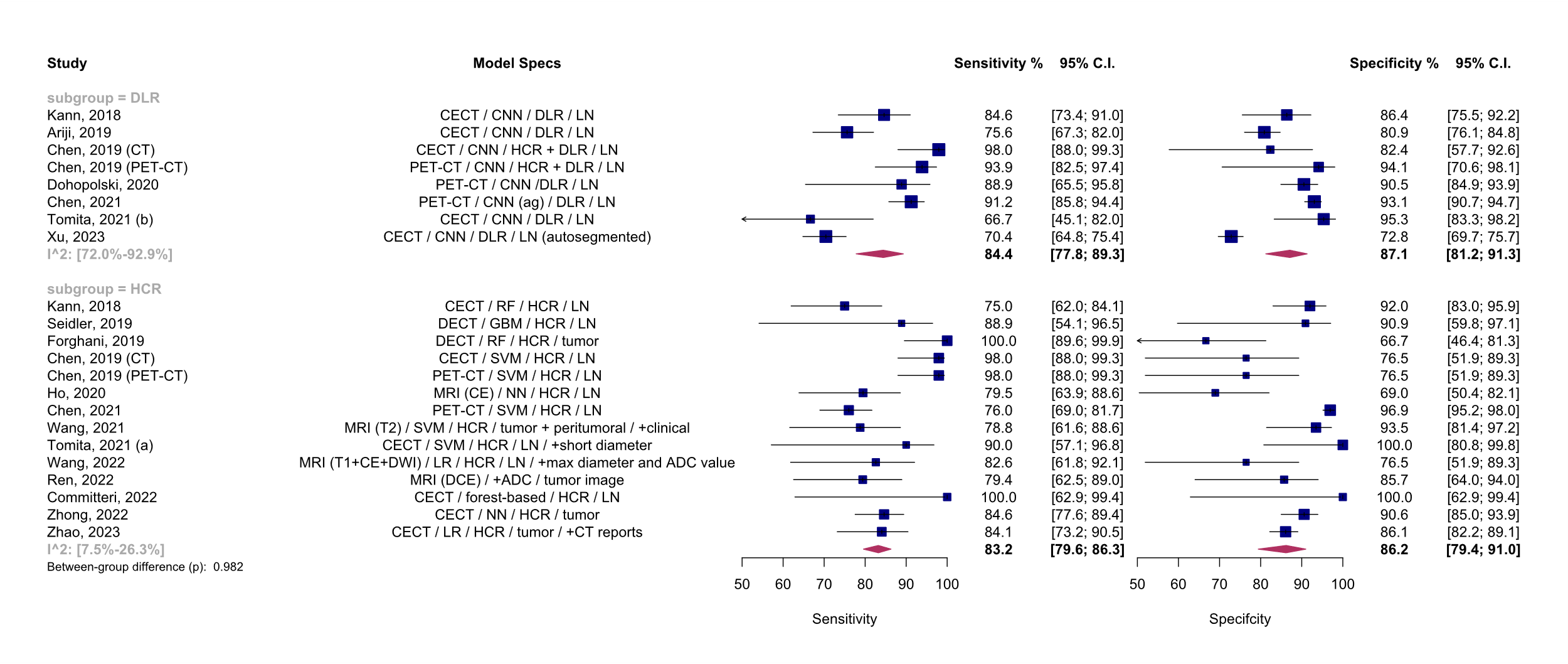


**Fig. A. 2** Paired Forest plots for the subgroup meta-analysis comparing models based on deep learning vs. hand-crafted radiomics, after excluding outlier studies, The between group difference is derived from bivariate model, ADC: apparent diffusion coefficient. ag: attention-guided. CE: contrast-enhanced. CECT: contrast-enhanced CT. CI: Confidence interval. CNN: Convolutional neural network. DCE: dynamic contrast-enhanced. DECT: dual energy CT. DLR: Deep learning radiomics. DWI: diffusion weighted imaging. GBM: Gradient Boosting Machine. HCR: hand-crafted radiomics. LN: lymph Node. LR: logistic regression. NB: naïve baYes. NN: neural network. RF: Random forest. SVM: Support vector machine


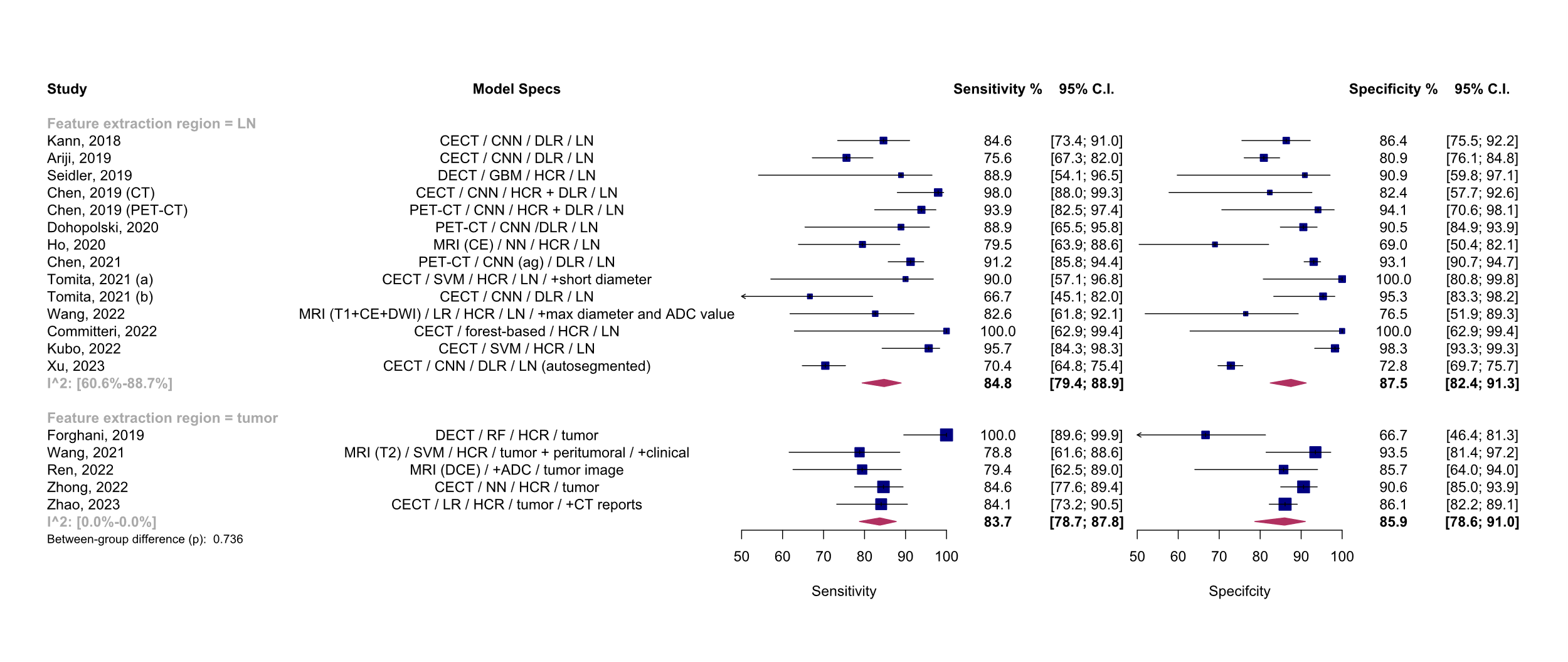


**Fig. A. 3** Paired Forest plots for the subgroup meta-analysis comparing models based on radiomics features extracted from lymph Nodes vs. primary tumor, after excluding one outlier study, The between group difference is derived from bivariate model, ADC: apparent diffusion coefficient. ag: attention-guided. CE: contrast-enhanced. CECT: contrast-enhanced CT. CI: Confidence interval. CNN: Convolutional neural network. DCE: dynamic contrast-enhanced. DECT: dual energy CT. DLR: Deep learning radiomics. DWI: diffusion weighted imaging. GBM: Gradient Boosting Machine. HCR: hand-crafted radiomics. LN: lymph Node. LR: logistic regression. NB: naïve baYes. NN: neural network. RF: Random forest. SVM: Support vector machine

| **Table Appendix 1. Results of the quality assessment of the included studies, based on METRICS scoring system.** | | | | | | | | | | | | | | | | | | | | | | | | | | | | |
| --- | --- | --- | --- | --- | --- | --- | --- | --- | --- | --- | --- | --- | --- | --- | --- | --- | --- | --- | --- | --- | --- | --- | --- | --- | --- | --- | --- | --- |
| **Items/Conditions** | **Definitions** | **Weights** | **Yuan 2021** | **Xu 2022** | **Konishi 2023** | **Wang 2022** | **Wang 2021** | **Kann 2019** | **Kann 2018** | **Tomita 2021** | **Tomita 2021** | **Lu 2022** | **Zhong 2022** | **Seidler 2019** | **Kubo 2022** | **Committeri 2022** | **Dohopolski 2020** | **Forghani 2019** | **Ho 2020** | **Chen 2019** | **Ariji 2021** | **Ariji 2019** | **Zhao 2023** | **Ariji (2)**  **2019** | **Bardosi 2022** | **Ren 2022** | **Chen 2021** | **Kudoh 2023** |
| **Study Design** | **Adherence to radiomics and/or machine learning-specific checklists or guidelines** | 0.0368 | Yes | Yes | Yes | Yes | Yes | Yes | Yes | Yes | Yes | Yes | Yes | Yes | Yes | Yes | Yes | Yes | Yes | Yes | Yes | Yes | Yes | Yes | Yes | Yes | Yes | Yes |
|  | **Eligibility criteria that describe a representative study population** | 0.0735 | Yes | Yes | Yes | Yes | Yes | Yes | Yes | Yes | Yes | Yes | Yes | Yes | Yes | Yes | Yes | Yes | Yes | Yes | Yes | Yes | Yes | Yes | Yes | Yes | Yes | Yes |
|  | **High-quality reference standard with a clear definition** | 0.0919 | Yes | Yes | Yes | Yes | Yes | Yes | Yes | Yes | Yes | Yes | Yes | Yes | Yes | Yes | Yes | Yes | Yes | Yes | Yes | Yes | Yes | Yes | Yes | Yes | Yes | Yes |
| **Imaging Data** | **Multi-center** | 0.0438 | No | No | No | No | No | Yes | Yes | No | No | No | No | No | No | No | No | No | No | No | No | No | No | No | No | No | No | No |
|  | **Clinical translatability of the imaging data source for radiomics analysis** | 0.0292 | Yes | Yes | No | Yes | Yes | Yes | Yes | Yes | Yes | Yes | Yes | Yes | Yes | Yes | Yes | Yes | Yes | Yes | Yes | Yes | Yes | Yes | Yes | Yes | Yes | Yes |
|  | **Imaging protocol with acquisition parameters** | 0.0438 | Yes | Yes | Yes | Yes | Yes | Yes | Yes | Yes | Yes | Yes | Yes | Yes | Yes | Yes | Yes | Yes | Yes | Yes | Yes | Yes | Yes | Yes | Yes | Yes | Yes | Yes |
|  | **The interval between imaging used and reference standard** | 0.0292 | Yes | Yes | Yes | Yes | Yes | Yes | Yes | Yes | Yes | Yes | Yes | Yes | Yes | Yes | Yes | Yes | Yes | Yes | Yes | Yes | Yes | Yes | Yes | Yes | Yes | Yes |
| **Segmentation** | **Does the study include segmentation?** | - | Yes | No | Yes | Yes | Yes | Yes | No | Yes | Yes | Yes | Yes | Yes | Yes | Yes | No | Yes | Yes | No | No | No | Yes | Yes | Yes | Yes | No | Yes |
|  | **Does the study include fully automated segmentation?** | - | No | No | Yes | No | No | No | No | No | No | No | No | No | Yes | No | No | No | No | No | No | No | No | No | No | No | No | No |
|  | **Transparent description of segmentation methodology** | 0.0337 | Yes | n/a | Yes | Yes | Yes | Yes | n/a | Yes | Yes | Yes | Yes | Yes | Yes | Yes | n/a | Yes | Yes | n/a | n/a | n/a | Yes | No | Yes | Yes | n/a | Yes |
|  | **Formal evaluation of fully automated segmentation** | 0.0225 | n/a | n/a | No | n/a | n/a | n/a | n/a | n/a | n/a | n/a | n/a | n/a | No | n/a | n/a | n/a | n/a | n/a | n/a | n/a | n/a | n/a | n/a | n/a | n/a | n/a |
|  | **Test set segmentation masks produced by a single reader or automated tool** | 0.0112 | Yes | n/a | Yes | Yes | Yes | Yes | n/a | Yes | Yes | Yes | Yes | Yes | Yes | No | n/a | Yes | Yes | n/a | n/a | n/a | Yes | Yes | No | Yes | n/a | Yes |
| **Image Processing and Feature Extraction** | **Does the study include hand-crafted feature extraction?** | - | No | No | No | No | No | No | No | No | No | No | No | No | No | No | No | No | No | No | No | No | No | No | No | No | No | No |
|  | **Appropriate use of image preprocessing techniques with transparent description** | 0.0622 | Yes | Yes | Yes | Yes | Yes | Yes | Yes | Yes | Yes | Yes | Yes | Yes | Yes | Yes | Yes | Yes | Yes | Yes | Yes | Yes | Yes | Yes | Yes | Yes | Yes | Yes |
|  | **Use of standardized feature extraction software** | 0.0311 | n/a | n/a | n/a | n/a | n/a | n/a | n/a | n/a | n/a | n/a | n/a | n/a | n/a | n/a | n/a | n/a | n/a | n/a | n/a | n/a | n/a | n/a | n/a | n/a | n/a | n/a |
|  | **Transparent reporting of feature extraction parameters, otherwise providing a default configuration statement** | 0.0415 | Yes | Yes | Yes | Yes | Yes | Yes | No | No | No | Yes | Yes | No | Yes | Yes | No | Yes | Yes | No | No | No | Yes | Yes | Yes | Yes | No | Yes |
| **Feature Processing** | **Does the study include tabular data?** | - | Yes | No | Yes | Yes | Yes | No | No | Yes | No | Yes | Yes | No | Yes | Yes | No | Yes | Yes | No | No | No | No | No | Yes | Yes | No | Yes |
|  | **Does the study include end-to-end deep learning?** | - | No | Yes | No | No | No | Yes | Yes | No | Yes | No | No | No | No | No | Yes | No | No | Yes | No | No | No | No | No | No | Yes | No |
|  | **Removal of Non-robust features** | 0.0200 | Yes | n/a | Yes | Yes | Yes | n/a | n/a | Yes | n/a | Yes | Yes | n/a | Yes | Yes | n/a | Yes | Yes | n/a | n/a | n/a | n/a | n/a | Yes | Yes | n/a | Yes |
|  | **Removal of redundant features** | 0.0200 | Yes | n/a | Yes | Yes | Yes | n/a | n/a | Yes | n/a | Yes | Yes | n/a | Yes | Yes | n/a | Yes | Yes | n/a | n/a | n/a | n/a | n/a | Yes | Yes | n/a | Yes |
|  | **Appropriateness of dimensionality compared to data size** | 0.0300 | Yes | n/a | Yes | Yes | Yes | n/a | n/a | Yes | n/a | Yes | Yes | n/a | Yes | Yes | n/a | Yes | Yes | n/a | n/a | n/a | n/a | n/a | Yes | Yes | n/a | Yes |
|  | **Robustness assessment of end-to-end deep learning pipelines** | 0.0200 | n/a | No | n/a | n/a | n/a | No | No | n/a | No | n/a | n/a | n/a | n/a | n/a | No | n/a | n/a | No | n/a | n/a | n/a | n/a | n/a | n/a | No | n/a |
| **Preparation for Modeling** | **Proper data partitioning process** | 0.0599 | Yes | Yes | Yes | Yes | Yes | Yes | Yes | Yes | Yes | Yes | Yes | Yes | Yes | Yes | Yes | Yes | Yes | Yes | Yes | No | Yes | Yes | Yes | Yes | Yes | Yes |
|  | **Handling of confounding factors** | 0.0300 | No | No | No | No | No | Yes | No | No | No | No | No | No | No | No | No | No | No | No | No | No | Yes | No | Yes | Yes | No | No |
| **Metrics and Comparison** | **Use of appropriate performance evaluation metrics for task** | 0.0352 | Yes | Yes | Yes | Yes | Yes | Yes | Yes | Yes | Yes | Yes | Yes | Yes | Yes | Yes | Yes | Yes | Yes | Yes | Yes | Yes | Yes | Yes | Yes | Yes | Yes | Yes |
|  | **Consideration of uncertainty** | 0.0234 | Yes | Yes | Yes | No | Yes | Yes | Yes | Yes | Yes | Yes | Yes | Yes | No | No | No | Yes | No | Yes | Yes | No | No | No | No | No | No | Yes |
|  | **Calibration assessment** | 0.0176 | Yes | Yes | Yes | Yes | Yes | Yes | Yes | No | Yes | Yes | Yes | Yes | No | Yes | Yes | No | Yes | Yes | Yes | No | Yes | Yes | Yes | Yes | Yes | Yes |
|  | **Use of uni-parametric imaging or proof of its inferiority** | 0.0117 | Yes | Yes | Yes | Yes | Yes | Yes | Yes | Yes | Yes | Yes | Yes | No | No | Yes | Yes | No | Yes | Yes | Yes | Yes | Yes | Yes | Yes | Yes | Yes | Yes |
|  | **Comparison with a Non-radiomic approach or proof of added clinical value** | 0.0293 | No | Yes | Yes | No | Yes | Yes | Yes | No | No | No | No | No | No | Yes | No | No | No | No | Yes | Yes | Yes | Yes | Yes | Yes | Yes | Yes |
|  | **Comparison with simple or classical statistical models** | 0.0176 | No | No | No | No | No | Yes | No | No | No | Yes | Yes | No | No | No | No | No | No | No | Yes | Yes | Yes | Yes | Yes | Yes | No | Yes |
| **Testing** | **Internal testing** | 0.0375 | No | Yes | Yes | No | No | Yes | Yes | Yes | Yes | Yes | Yes | Yes | No | No | Yes | Yes | No | Yes | Yes | No | Yes | Yes | No | No | No | No |
|  | **External testing** | 0.0749 | No | No | No | No | No | Yes | No | No | No | No | No | No | No | No | No | No | No | No | No | No | No | No | No | No | No | No |
| **Open Science** | **Data availability** | 0.0075 | No | No | No | Yes | No | No | No | No | No | Yes | No | No | No | No | No | No | No | No | No | No | No | No | No | No | No | No |
|  | **Code availability** | 0.0075 | No | No | No | No | No | No | No | No | No | No | Yes | No | No | No | No | No | No | No | No | No | No | No | No | No | No | Yes |
|  | **Model availability** | 0.0075 | No | No | No | No | No | No | No | No | No | No | No | No | No | No | No | No | No | No | No | No | No | No | No | No | No | Yes |
| **Total METRICS score:** | | | **72.8%** | **74.9%** | **75%** | **71%** | **75.8%** | **95.2%** | **76.7%** | **70.1%** | **68.1%** | **79.2%** | **79.8%** | **68.3%** | **65.4%** | **72.2%** | **63.6%** | **73.3%** | **70.2%** | **66.4%** | **73.8%** | **58.1%** | **80.8%** | **73.3%** | **77.3%** | **78.5%** | **63.4%** | **79.4%** |
| **Quality category:** | | | **Good** | **Good** | **Good** | **Good** | **Good** | **Excellent** | **Good** | **Good** | **Good** | **Good** | **Good** | **Good** | **Good** | **Good** | **Good** | **Good** | **Good** | **Good** | **Good** | **Moderate** | **Excellent** | **Good** | **Good** | **Good** | **Good** | **Good** |
